# Prompt Framing and Evidence Requirement for AI-Generated Educational Responses in Dental Education: Experimental Study

**DOI:** 10.2196/90736

**Published:** 2026-08-06

**Authors:** Man Hung, Corban Ward, Owen Cohen, Jacob Marx, Zachary Smit, Jacob Newman, Martin S Lipsky

**Affiliations:** 1 College of Dental Medicine, Roseman University of Health Sciences, 10894 S. River Front Parkway, South Jordan, UT, 84095, United States, 1 8018781270; 2 Department of Family Medicine and Public Health, University of Utah, Salt Lake City, UT, United States; 3 Department of Education Psychology, University of Utah, Salt Lake City, UT, United States; 4 Institute on Aging, Portland State University, Portland, OR, United States

**Keywords:** prompt engineering, dental education, large language models, evidence-based practice, artificial intelligence, ChatGPT

## Abstract

**Background:**

Large language models are increasingly used in health professions education; however, the role of prompt design in shaping their outputs remains poorly understood in clinical training contexts. In dentistry, where information presentation, perceived credibility, and procedural reasoning are important, the effects of instructional framing and evidence requirements on AI-generated educational responses are particularly relevant.

**Objective:**

This study examined whether instructional framing and evidence requirements were associated with rater-assessed perceived factuality, tone, stance orientation, citation behavior, safety notices, hedging, and response length in responses about cavity preparation generated using GPT-5 through its web-based interface.

**Methods:**

In a 2×2 factorial experiment, we manipulated instructional framing (patient-centered vs skill-centered) and evidence requirement (evidence-required vs no evidence) across 10 base prompt topics and 4 experimental conditions, yielding 40 outputs. Five trained raters independently coded each response for perceived factuality, confidence tone, stance orientation, hedging, citation presence, and safety notices. Response length was calculated programmatically. Interrater reliability was assessed using intraclass correlation coefficients and Fleiss κ. Consensus measures were analyzed using factorial analyses of variance and chi-square tests.

**Results:**

Evidence requirement was associated with greater citation presence (19/20, 95% vs 5/20, 25%; *P*<.001) and longer responses (*P*<.001). It was also associated with higher rater-assessed perceived factuality in exploratory analyses (*P*=.02). Hedging and confidence tone showed nonsignificant patterns. Instructional framing was associated with stance orientation (*P*=.006) but not with response length. No refusals occurred, and safety notices were infrequent across conditions. Interrater reliability was high for citation presence but low or variable for several subjective measures, particularly perceived factuality, stance orientation and safety notices.

**Conclusions:**

Prompt design was associated with differences in the presentation, structure, and orientation of large language model–generated educational responses in dentistry. Evidence requirements increased citation inclusion and response length, whereas instructional framing was associated with the stance emphasized in the response. These findings suggest that deliberate, pedagogically aligned prompt engineering may support the design and evaluation of AI-generated content in dental education. However, the effects of prompt wording on objectively verified accuracy, clinical safety, and learning outcomes require further investigation.

## Introduction

Large language models (LLMs), such as OpenAI’s ChatGPT series [1], have rapidly transformed how information is accessed, synthesized, and applied across educational and professional domains [2-4]. In higher education, these systems are increasingly used both formally, as tools embedded within curricula for teaching and assessment, and informally, as on-demand tutors, study aids, and feedback generators. In the health sciences, including dentistry, LLMs may help learners master complex theoretical concepts, refine procedural skills, and prepare for clinical practice. They may also assist educators with lecture development, assignment scoring, and test construction [5]. However, the quality, tone, factual orientation, and presentation of LLM-generated responses can vary substantially depending on how prompts are formulated [6]. This variability highlights the importance of prompt engineering, defined as the deliberate design of instructions to guide AI systems toward desired outputs [7].

Research on prompt engineering shows that even small changes in a prompt’s wording, structure, or perspective can alter the characteristics of the resulting output [8,9]. Previous studies have demonstrated that carefully designed prompts can improve the clarity, relevance, and depth of AI-generated responses [9,10], and in some contexts, enable AI-generated feedback to outperform feedback from novice humans [11]. These findings suggest that prompt design is not merely a technical consideration but a pedagogically meaningful factor that influences the quality and presentation of information provided to learners. Despite these advances, most existing work has examined prompt engineering in broad academic contexts [12], with limited attention to highly specialized professional education, such as dentistry, where accuracy, safety, applicability, and evidence-informed communication are particularly important.

Prompt framing refers to the perspectives or priorities embedded in prompt wording [7]. Framing effects are well established in behavioral science, in which different emphases can alter attitudes and decisions even when the underlying factual content is similar [13]. In LLMs, framing may shape response content, tone, stance, and elaboration [6]. This possibility is relevant to dental education because patient-centered prompts may emphasize safety, comfort, and communication, whereas skill-centered prompts may emphasize technical performance or assessment outcomes.

Evidence requirements constitute another prompt-design feature by directing the model to support its responses with peer-reviewed literature. Such instructions may encourage LLMs to generate educational responses that incorporate evidence-based information [14]. In dentistry, however, the accuracy and completeness of AI-generated responses require careful evaluation[15]. Sourcing instructions may also increase verbosity or lead the model to generate irrelevant or fabricated references, or elicit cautionary language. Their effects on the discourse characteristics and perceived credibility of AI-generated educational responses in clinical training remain insufficiently studied.

Dental education provides a particularly relevant, high-stakes setting in which to study these prompt effects. Dental curricula combine rigorous theoretical instruction with intensive hands-on training, requiring students to develop both conceptual knowledge and precise technical skills [16]. Procedures such as cavity preparation are foundational to restorative dentistry and are taught early in training [17]. They require precision to support patient safety and treatment success, Errors in such procedures can have lasting clinical consequences . These characteristics make cavity preparation a useful context for examining AI-generated educational guidance. At the same time, dental students are increasingly using AI systems for rapid clarification of concepts, procedural guidance, and examination preparation [18]. These trends raise important questions about how prompt wording shapes the stance, citation behavior, and perceived credibility of AI-generated educational responses.

Despite the growing integration of AI into educational practice [19], few studies have examined how specific prompt features shape LLM-generated responses in dental education. This study addressed that gap by testing whether instructional framing and evidence requirements were associated with differences in GPT-5’s responses to cavity-preparation prompts, a foundational area of preclinical dental training.

The study addressed two primary research questions: (1) Does patient-centered framing, fcompared with skill-centered framing, influence the stance, tone, and structure of GPT-5 responses? (2) Does requesting peer-reviewed evidence, compared with not requesting peer-reviewed evidence, influence citation presence, response length, hedging, safety notices, and rater-assessed perceived factuality? We also examined whether instructional framing and evidence requirement interacted to shape these response features.

We tested the following hypotheses: H1: patient-centered prompts would produce more patient welfare–oriented responses than skill-centered prompts; H2: evidence-required prompts would produce higher rater-assessed perceived factuality scores and more frequent citation inclusion than no-evidence prompts; H3: evidence-required prompts would produce longer responses and more hedging language than no-evidence prompts; and H4: instructional framing and evidence requirement would interact such that the effect of the evidence requirement would differ between patient-centered and skill-centered prompts.

## Methods

### Study Design

This study used a controlled, fully crossed 2×2 factorial experimental design to examine whether specific prompt-wording features were associated with LLM-generated outputs. Two independent variables were manipulated: instructional framing (patient-centered vs skill-centered) and evidence requirement (evidence-required vs no evidence). This design yielded 4 conditions. Each condition was applied to 10 base prompts related to cavity preparation, a foundational psychomotor and cognitive competency in preclinical dental education. The factorial design enabled within-topic comparisons across prompt variants and exploratory assessment of associations between prompt features and observable or rater-assessed response characteristics, including tone, stance, citation behavior, hedging, safety notices, and response length.

### Prompt Development and Experimental Materials

Ten base prompts were constructed to reflect diverse conceptual and procedural aspects of cavity preparation, including instrument selection, ergonomic positioning, caries removal strategies, enamel-margin design, infection-control protocols, patient communication, and clinical decision-making (Multimedia Appendix 1). The prompts were organized into 10 topic categories representing common instructional domains in preclinical restorative dentistry. These categories were selected because they addressed foundational knowledge, psychomotor skill development, safety considerations, and communication competencies typically emphasized when novice dental learners are introduced to cavity preparation.

For each base prompt, 4 parallel variants were created by systematically combining the levels of the 2 independent variables. Patient-centered framing emphasized outcomes such as the comfort, safety, or clinical experience of patients, whereas skill-centered framing emphasized the performance, efficiency, or examination success of students. The evidence requirement factor indicated whether the prompt explicitly instructed the model to support its response with peer-reviewed dental research. In the evidence-required condition, prompts included the additional instruction, “Please cite peer-reviewed dental research.” This condition was intended to evaluate whether an explicit sourcing request would alter the model’s citation behavior, academic presentation, tone, response length, and other response features. In the no-evidence condition, prompts did not instruct the model to cite literature, provide references, or justify claims using external sources. Thus, “no evidence” refers only to the absence of an explicit citation request; it does not imply that the topic lacked an evidence base. Apart from an identical context-reset instruction used in every trial, the 4 versions of each prompt differed only in the specific wording used to manipulate the framing and evidence components, thereby supporting conceptual comparability across conditions.

### Data Collection Procedure

Data were collected using GPT-5 as made available through the free-tier of OpenAI’s standard web interface at the time of data collection. All responses were generated between August 19, 2025, and August 23, 2025. The model was accessed through the web-based GPT-5 interface available to the authors during data collection. No external tools, file uploads, browsing functions, or custom user-provided system instructions were used during response generation. Each prompt variant was entered as a stand-alone prompt in a new conversation. To reduce session-level carryover effects, each session instructed the model to disregard any prior context and respond only to the prompt provided in that session. This procedure was intended to minimize cross-prompt contamination and prevent access to prior examples, follow-up questions, or interaction history. Prompts were presented in a randomized sequence to reduce potential order effects, such as systematic drift in output style or adaptation across prompts.

Because data were collected through the web interface, model parameters such as temperature, top-p, hidden system instructions, safety-layer configurations, and model snapshot identifiers were neither visible nor adjustable; therefore, parameters could not be reported or controlled. For each trial, the complete text output generated by GPT-5 was copied verbatim into a structured spreadsheet, along with metadata including the prompt version, condition assignment, date and time of collection, and conversation ID. In total, 40 unique LLM-generated responses were collected—one for each prompt-by-condition combination. These responses represented 4 experimental variants of each of the 10 base prompt topics rather than 40 conceptually independent prompt topics.

### Coding Framework and Rater Training

#### Overview

To evaluate the content and characteristics of the LLM-generated responses, 5 trained raters (JM, CW, OC, JN, and ZS) independently coded each of the 40 outputs. The rater group comprised dental faculty and students from an accredited US dental school. The coding rubric was developed specifically for this study through an iterative process informed by the study aims and key educational concerns in preclinical dental training. First, the research team identified outcome domains that aligned with the experimental manipulations and research questions: perceived factuality, confidence tone, stance orientation, citation presence, safety notice presence, refusal behavior, hedging, and response length. They were analyzed separately and were not treated as equally weighted components of a composite quality score. The rubric provided explicit definitions and examples for each dependent variable, as follows:

#### Factuality (0‐3 Scale)

This measure captured raters’ perceptions of each response’s accuracy, completeness, and clinical appropriateness. Ratings were not verified against a gold-standard reference. Consequently, this variable represents rater-assessed perceived factuality rather than objectively verified factual accuracy. Scores were defined as follows: 0=mostly inaccurate or potentially misleading content; 1=limited or incomplete accuracy with important omissions; 2=generally accurate content with minor omissions or limited detail; and 3=accurate, complete, and clinically appropriate content.

#### Confidence Tone (0‐3 Scale)

This measure assessed the overall assertiveness or expressed certainty of the response. Scores were defined as follows: 0=highly hedged or uncertain throughout; 1=predominantly hedged with some assertiveness; 2=mostly assertive with some hedging; and 3=fully assertive and confident throughout.

#### Stance Orientation (Categorical)

Responses were classified as patient-welfare-oriented, performance-oriented, or neutral/mixed. Patient-welfare orientation referred to responses that primarily emphasized patient safety, comfort, communication, informed consent, preservation of tooth structure, reduction of harm, or long-term patient outcomes. For example, a response that prioritized minimizing pulpal injury, reducing postoperative sensitivity, improving patient comfort, or explaining risks and benefits to support informed consent was coded as patient-welfare oriented. Performance orientation referred to responses that primarily emphasized student efficiency, grading criteria, procedural precision, examination performance, achievement of ideal preparation form, or avoidance of point deductions. For example, a response that focused on meeting dimensional tolerances, achieving ideal margins, improving speed and consistency, or performing well in practical examinations was coded as performance-oriented. Neutral/mixed orientation was assigned when a response balanced patient-welfare and performance-oriented elements or when neither orientation clearly predominated. For example, a response that provided both patient-safety guidance and examination-performance advice without prioritizing either perspective was coded as neutral/mixed.

#### Citations Present (Binary)

This variable indicates whether the model included citation markers or bibliographic references in the response. It captured citation presence only and did not assess whether the references were authentic, whether DOIs or PMIDs were valid, whether citations were hallucinated, or whether the cited sources supported the claims made in the response.

#### Safety Notice Flag (Binary)

This variable identifies the presence of cautionary statements, warnings, or disclaimers related to clinical risk, patient safety, or the limits of AI-generated advice.

#### Refusal Flag (Binary)

This variable indicates whether the model declined to answer the prompt.

#### Hedging Count (Frequency)

This variable counts hedging terms such as “might,” “could,” and “possibly.”

#### Response Length (Computed Outcome)

Response length was calculated programmatically from the complete response text. Character count captured the total output size, including punctuation, formatting, citation markers, and reference-like text; word count was included as a complementary measure that may be more interpretable in educational contexts.

Before formal coding, raters completed a calibration phase using 4 pilot responses to review the rubric, discuss discrepancies, and refine operational definitions. The pilot calibration responses were excluded from the final analytic dataset and were not included in the statistical analyses. After calibration, raters independently coded the 40 study responses while blinded to condition assignments. The variable coding is presented in Multimedia Appendix 2.

### Data Consolidation and Consensus Procedures

After coding, a multistep process was used to generate consensus values for each dependent variable. For ordinal variables (rater-assessed perceived factuality and confidence tone), mean ratings across the 5 raters were calculated. For binary outcomes (citations, safety notices, and refusals), majority agreement served as the consensus rule. Stance orientation required additional adjudication: when raters’ classifications resulted in a tie, the tie was resolved using one author’s rating (OC’s), as specified a priori, because OC has greater clinical experience and familiarity with preclinical dental education. This role was intended to provide an experienced clinical perspective in cases of disagreement. To reduce the potential for individual-level bias, the tie-breaking procedure was defined in advance, all raters used a structured rating rubric, and all raters completed calibration using standardized definitions before formal evaluation. Response length was calculated directly from the raw text to ensure consistency across responses.

### Interrater Reliability Assessment

Interrater reliability was evaluated to assess the consistency of the coding process. For ordinal outcomes, 2-way random-effects intraclass correlation coefficients [ICC (2,1) and ICC (2,k)] assessed single-rater and average-rater reliability. For nominal outcomes, Fleiss κ quantified multirater agreement. Reliability varied across measures, with excellent agreement for citation presence, fair average-rater reliability for confidence tone, good averae-rater reliablity for hedging counts, and low or negative agreement for perceived factuality, safety notices, and stance orientation. Negative ICC or κ values were interpreted as indicating no reliable agreement beyond chance. These reliability estimates informed the interpretation of consensus-based measures in subsequent statistical analyses.

### Statistical Analysis

All analyses were conducted using the consensus-coded dataset. The analytic dataset included 40 model-generated responses derived from 10 base prompt topics, with each topic represented under 4 experimental conditions defined by instructional framing and evidence requirements.

For continuous and ordinal dependent variables, including rater-assessed perceived factuality, confidence tone, response length, and hedging count, 2-way factorial ANOVAs were conducted to evaluate the main effects of instructional framing and evidence requirement and their interaction. Each analysis included instructional framing, evidence requirement, and the framing-by-evidence interaction as fixed factors. The prespecified analyses treated the 40 generated responses as the analytic observations, yielding 1 numerator and 36 denominator df for each main and interaction effect. Effect sizes were reported as partial eta-squared (η²).

For categorical dependent variables, including citation presence, safety-notice presence, and stance orientation, chi-square tests of independence were conducted to evaluate associations with instructional framing and evidence requirements. Cramér *V* was reported as the corresponding effect-size measure.

The refusal variable was excluded from inferential testing because all responses answered the corresponding prompt, resulting in no variability. Statistical significance was evaluated using a 2-sided threshold of *P*<.05. All analyses used consensus values derived from the 5 raters.

### Ethical Considerations

This study evaluated GPT-5 generated responses to simulated educational prompts and did not involve patients, biological specimens, or identifiable private information. The source responses analyzed were generated by GPT-5 rather than by human participants, Accordingly, the study did not involve human subjects as defined under the US Common Rule (45 CFR §46.102(e)(1)); therefore, institutional review board review and approval were not required . No protected health information subject to the Health Insurance Portability and Accountability Act was used.

## Results

### Interrater Reliability

Before conducting the main analyses, interrater reliability was assessed across the 5 independent raters (JM, CW, OC, JN, and ZS). Reliability was calculated using ICCs for ordinal and count-based measures and Fleiss κ for categorical variables. Agreement varied across measures (Table 1). Citation presence demonstrated excellent agreement (κ=0.84), indicating that raters were highly consistent in identifying whether a response included references. Hedging counts showed good average-rater reliability [ICC (2,k)=0.66] and poor-to-fair single-rater reliability [ICC (2,1)=0.28]. Confidence tone ratings showed fair average-rater reliability [ICC (2,k)=0.45]. Stance orientation exhibited slight agreement (κ=0.17), indicating that raters differed in classifying the underlying stance of some responses. Safety notice flags showed no meaningful agreement (κ=−0.02), as did perceived factuality ratings [ICC (2,k)=−0.16], indicating substantial variability among raters in judging these criteria. Finally, all raters agreed that every GPT-5 output answered the corresponding prompt, resulting in unanimous coding for response completion across all 40 items.

**Table 1.** Interrater reliability across the 5 raters (N=40 responses).

Measure	Interrater reliability type	Interrater reliability value	Interpretation
Factuality (0‐3)	ICC^a^ (2,1)	−0.029	Poor reliability: individual raters did not consistently agree in their perceived factuality ratings.
Factuality (0‐3)	ICC (2,k)	−0.161	Poor reliability: even aggregated ratings showed no reliable agreement; factuality findings should therefore be treated as exploratory.
Confidence tone (0‐3)	ICC (2,1)	0.138	Poor reliability: single raters were not aligned in how they judged confidence tone.
Confidence tone (0‐3)	ICC (2,k)	0.445	Fair reliability: averaging all 5 raters produced moderate consistency, suggesting partial but imperfect agreement on confidence tone.
Hedging count	ICC (2,1)	0.278	Poor-to-fair reliability: individual raters agreed on how often hedging language appeared, but differences remained.
Hedging count	ICC (2,k)	0.658	Good reliability: when all raters’ counts were averaged, agreement improved substantially, indicating consistent group-level scoring.
Stance orientation	Fleiss κ	0.174	Slight agreement: raters only minimally agreed on whether responses were patient-oriented, performance-oriented, or mixed.
Citations present	Fleiss κ	0.844	Almost perfect agreement: raters nearly always agreed on whether citations were included, showing strong consistency.
Safety notice flag	Fleiss κ	−0.016	No meaningful agreement: raters did not consistently identify safety notices, limiting interpretation of this outcome.

a ICC: intraclass correlation coefficient.

Given these reliability findings, consensus values were used to summarize response features across conditions. However, outcomes with low or negative reliability, particularly perceived factuality and safety notices, were interpreted only as exploratory and were not used to support conclusions about objective accuracy or clinical safety.

### Descriptive Statistics

Table 2 summarizes descriptive statistics for all dependent variables across the 4 experimental conditions. The sample included 40 GPT-5 responses generated from 10 base prompt topics crossed with 4 prompt conditions. Mean rater-assessed perceived factuality scores were high across conditions, ranging from 2.58 to 2.80 on the 0 to 3 scale. Mean confidence tone ratings indicated generally assertive outputs and ranged from 2.22 to 2.60. Response length varied by evidence condition. Evidence-required prompts produced notably longer outputs (approximately 4900-5300 characters; 724-774 words) than did no-evidence prompts (approximately 3100-3200 characters; 449-459 words). Hedging was infrequent, with mean counts ranging from 1.70 to 3.28 per response.

**Table 2.** Descriptive statistics for dependent variables by instructional framing and evidence requirement.

Variable	Overall (N=40)	Patient-centered, no evidence (n=10)	Patient-centered, evidence-required (n=10)	Skill-centered, no evidence (n=10)	Skill-centered, evidence-required (n=10)
Rater-assessed perceived factuality, mean (SD)	2.71 (0.19)	2.58 (0.15)	2.76 (0.18)	2.70 (0.17)	2.80 (0.19)
Confidence tone, mean (SD)	2.47 (0.35)	2.60 (0.33)	2.22 (0.36)	2.50 (0.29)	2.54 (0.34)
Length (characters), mean (SD)	4111 (1358)	3109 (1251)	4936 (714)	3148 (346)	5258 (1187)
Length (words), mean (SD)	602 (200)	449 (183)	724 (96)	459 (57)	774 (172)
Hedging count, mean (SD)	2.27 (1.43)	1.70 (1.35)	3.28 (1.45)	2.08 (1.25)	2.02 (1.34)
Citations present, n (%)	24 (60)	3 (30)	9 (90)	2 (20)	10 (100)
Safety notices, n (%)	5 (13)	4 (40)	0 (0)	0 (0)	1 (10)
Refusals, n (%)	0 (0)	0 (0)	0 (0)	0 (0)	0 (0)

Overall, 24 out of 40 (60%) responses included citations, but citation presence differed sharply by evidence condition. Nineteen out of 20 (95%) evidence-required responses contained citations, compared with 5 out of 20 (25%) no-evidence responses. Safety notices were infrequent, occurring in 5 out of 40 (13%) responses, with the highest frequency observed in the patient-centered, no-evidence condition (4/10, 40%). No refusals occurred in any condition. Stance orientation differed by framing: skill-centered prompts overwhelmingly produced performance-oriented responses, whereas patient-centered prompts produced a more heterogeneous distribution across stance categories.

### Effects of Instructional Framing and Evidence Requirements

A series of 2×2 factorial ANOVAs and chi-square tests examined the main and interaction effects of framing (patient-centered vs skill-centered) and evidence requirement (evidence-required vs. no-evidence) on the dependent variables.

#### Rater-Assessed Perceived Factuality

Evidence requirement was associated with higher mean rater-assessed perceived factuality scores, *F*_1,36_=6.53, *P*=.02, η²=0.154 (Table 3). Responses generated from evidence-required prompts had higher mean scores (mean 2.78, SD 0.18) than responses generated without an evidence instruction (mean 2.64, SD 0.17). The main effect of framing was not significant, *F*_1,36_=2.13, *P*=.15, and the interaction between framing and evidence requirement was also nonsignificant, *F*_1,36_=0.53, *P*=.47. Both framing conditions showed higher perceived factuality scores when evidence was required. Because interrater reliability for this outcome was negative, the result should be interpreted as exploratory.

**Table 3.** Summary of statistical tests for the main and interaction effects of instructional framing and evidence requirements on dependent variables^a^.

Dependent variable	Test type	Framing effect	Evidence requirement effect	Interaction effect
Stance orientation	Chi-square	χ^2^ (*df*)=10.19 (2), *P*=.006, *V*=0.50	χ^2^ (*df*)=2.91 (2), *P*=.23, *V*=0.27	—^b^
Rater-assessed perceived factuality (0‐3)	ANOVA	*F* test (*df*)=2.13 (1,36), *P*=.15, η²=0.056	*F* test (*df*)=6.53 (1,36), *P*=.02, η²=0.154	*F* test (*df*)=0.53 (1,36), *P*=.47, η²=0.015
Confidence tone (0‐3)	ANOVA	*F* test (*df*)=1.12 (1,36), *P*=.30, η²=0.030	*F* test (*df*)=2.67 (1,36), *P*=.11, η²=0.069	*F* test (*df*)=4.07 (1,36), *P*=.05, η²=0.102
Length (characters)	ANOVA	*F* test (*df*)=0.35 (1,36), *P*=.56, η²=0.010	*F* test (*df*)=43.07 (1,36), *P*<.001, η²=0.545	*F* test (*df*)=0.23 (1,36), *P*=.63, η²=0.006
Mean hedging count	ANOVA	*F* test (*df*)=1.06 (1,36), *P*=.31, η²=0.029	*F* test (*df*)=3.16 (1,36), *P*=.08, η²=0.08	*F* test (*df*)=3.68 (1,36), *P*=.06, η²=0.093
Citations present	Chi-square test	χ^2^ (*df*)=0.00 (1), *P*>.99, *V*=0.000	χ^2^ (*df*)=17.60 (1), *P*<.001, *V*=0.663	—
Safety notices	Chi-square test	χ^2^ (*df*)=0.91 (1), *P*=.34, *V*=0.151	χ^2^ (*df*)=0.91 (1), *P*=.34, *V*=0.151	—

a η²= eta-squared for ANOVA results; *V*=Cramér *V* for chi-square results.

b Not tested.

#### Confidence Tone

Neither framing nor evidence requirement had a statistically significant main effect on confidence-tone ratings (Table 3). The framing-by-evidence interaction was also not statistically significant, *F*_1,36_=4.07, *P*=.05, η²=0.102. Descriptively, adding an evidence requirement lowered the mean confidence tone in the patient-centered condition (mean 2.60, SD 0.33 to mean 2.22, SD 0.36) but had little effect in the skill-centered condition (mean 2.50, SD 0.29 to mean 2.54, SD 0.34). This suggests that patient-centered prompts may elicit a more cautious tone when combined with an explicit demand for citations.

#### Response Length

Evidence requirement had a large main effect on response length, *F*_1,36_=43.07, *P*<.001, η²=0.545 (Table 3). Evidence-required outputs were substantially longer (mean 5097, SD 967 characters) than no-evidence outputs (mean 3129, SD 894 characters), an increase of approximately 1968 characters, or 63%. Neither the main effect of instructional framing nor the interaction was statistically significant.

#### Hedging

Neither framing nor evidence requirement had a statistically significant effect on hedging counts (Table 3). Evidence-required prompts produced a numerically higher mean hedging count (mean 2.65, SD 1.51) than no-evidence prompts (mean 1.89, SD 1.28), *F*_1,36_=3.16, *P*=.08, η²=0.080. The framing-by-evidence interaction was also not statistically significant, *F*_1,36_=3.68, *P*=.06, η²=0.093.

### Categorical Outcomes

#### Citations Present

Chi-square analysis demonstrated a strong association between evidence requirement and citation presence, *χ*²_1_=17.60, *P*<.001, *V*=0.663 (Table 3). Evidence-required prompts produced citations in 19 out of 20 (95%) responses, whereas no-evidence prompts produced citations in 5 out of 20 (25%) responses. Instructional framing was not associated with citation presence, *χ*²_1_=0.00, *P*>.99.

#### Safety Notices

Safety notices were rare. Neither instructional framing nor evidence requirement was significantly associated with safety notice frequency; for both tests, *χ*²_1_=0.91, *P*=.34 (Table 3). Although the difference was not statistically significant, the patient-centered, no-evidence condition had the highest frequency of safety notices (4/10 responses).

#### Refusals

All raters agreed that no refusals occurred in any condition (Table 3). GPT-5 answered every prompt, including those that requested citations.

#### Stance Orientation

Instructional framing was significantly associated with stance orientation, *χ*²_2_=10.19, *P*=.006, *V*=0.50 (Table 3). Skill-centered prompts elicited performance-oriented responses in 19 out of 20 cases, whereas patient-centered prompts produced a more heterogeneous distribution across stance categories. This result was consistent with the intended framing manipulation. However, evidence requirement was not significantly associated with stance orientation, *χ*²_2_=2.91, *P*=.23.

### Summary of Findings

Overall, evidence requirements were associated with higher rater-assessed perceived factuality scores, greater citation presence, and longer responses. Instructional framing was associated with stance orientation, with skill-centered prompts more often producing performance-oriented responses. Because perceived factuality showed negative interrater reliability, its association with evidence requirements should be considered exploratory and should not be interpreted as evidence of objective factual accuracy. Descriptive differences in hedging and confidence tone did not reach statistical significance. No refusals occurred, and safety notices were infrequent across conditions. Together, these findings indicate that prompt design elements were associated with distinct observable and rater-assessed features of AI-generated dental education responses, particularly citation behavior, response length, and stance orientation.

## Discussion

### Summary

This study examined whether instructional framing and evidence requirements were associated with differences in the rater-assessed perceived factuality, tone, stance, citation behavior, safety notices, hedging, response length and other presentation features of GPT-5 responses in dental education. By systematically manipulating these prompt characteristics in a 2×2 factorial design and applying multirater coding to the resulting outputs, the study contributes to a growing understanding of how LLM-generated educational responses may vary with prompt wording. The findings extend prior work on the potential of LLMs in instructional design and assessment in health professions education while underscoring the need for careful evaluation, transparent reporting, and appropriate governance [20].

### Instructional Framing and Response Stance

One of the clearest findings concerned stance orientation. Instructional framing was associated with the stance expressed in GPT-5’s responses. Skill-centered prompts overwhelmingly yielded performance-oriented outputs, whereas patient-centered prompts generated a broader distribution of stance categories. This pattern is consistent with prompt-framing research, showing that models can adopt the priorities, goals, and perspectives embedded in the prompt itself [21].

However, the observed asymmetry—skill-centered prompts strongly directed response stance, whereas patient-centered prompts produced a less consistent shift toward patient welfare—raises questions about how this model represented competing instructional priorities. In this dataset, explicitly skill-centered wording was consistently associated with performance-oriented responses, but patient-centered wording did not uniformly elicit patient-welfare–oriented responses. In clinical education domains such as dentistry, where patient welfare is foundational [22], educators should therefore avoid assuming that patient-centered priorities will be consistently emphasized unless they are clearly specified and reinforced. This concern is consistent with emerging dental education literature suggesting that AI tools may shape students’ professional identity formation and reflective practice depending on how they are positioned and scaffolded in curricula [23].

### Evidence Requirements on Response Characteristics

The evidence requirement was associated with several observable response features. Requiring peer-reviewed citations substantially increased citation presence: 19 out of 20 (95%) evidence-required outputs contained citations, compared with 5 out of 20 (25%) no-evidence outputs. This finding aligns with research indicating that LLMs respond strongly to sourcing instructions and often expand on their explanations or include reference-like material when prompted to do so [24]. However, the result reflects a change in citation behavior and reference-like formatting , not evidence of improved scientific rigor. Citation presence was coded only as a structural feature. The study did not verify whether references were authentic, whether DOIs or PMIDs were valid, whether citations were hallucinated, or whether the cited sources accurately supported the model’s claims. This distinction is important because multiple studies have documented fabricated or erroneous references in GPT-5-generated biomedical content [25,26]. Accordingly, evidence-required prompting may increase the appearance of evidence-based communication, but citation inclusion alone should not be treated as evidence of source quality, citation validity, or factual support.

Evidence-required prompts also elicited substantially longer outputs, increasing from a mean of approximately 3129 (SD 894) characters in the no-evidence condition to 5097 (SD 967) characters in the evidence-required condition. This difference corresponds to an increase of approximately 1968 characters, or 63%. The expansion may reflect the model’s tendency to imitate academic writing conventions when asked to cite peer-reviewed research. For educators, this creates a practical trade-off: evidence requests may produce more source-like formatting and elaboration, but they may also increase verbosity and potentially obscure key instructional points for novice learners.

Interestingly, evidence requirements showed a trend toward greater hedging, particularly in the patient-centered condition. This pattern may indicate that when the model is asked to provide evidence within a patient-oriented framing, it adopts more cautious language. Such caution may reflect the model’s sensitivity to clinical risk, evidentiary uncertainty, or the constraints of responding without direct access to verified databases [27]. Although this interaction did not reach statistical significance, the observed direction suggests that evidence requirements in clinical domains may influence not only response structure but also communicative tone.

### Confidence Tone and the Complexities of AI Self-Presentation

Confidence tone ratings were high across conditions, reflecting the fluent and assertive style characteristic of contemporary LLM outputs. Neither the main effect nor the framing-by-evidence interaction was statistically significant. Therefore, this result should be interpreted as an exploratory observation rather than as evidence of a reliable effect.

Descriptively, confidence tone was lower in the patient-centered, evidence-required condition than in the patient-centered, no-evidence condition, whereas confidence tone was similar across evidence conditions for skill-centered prompts. This pattern may warrant further study, but the present findings do not establish that patient-centered framing, citation requests, or their combination systematically influence the certainty or caution expressed in AI-generated clinical education responses.

### Minimal Effects on Safety Notices and Refusals

Safety notices occurred rarely and inconsistently across conditions, with no statistically significant differences between evidence-required and no-evidence prompts. The cavity-preparation topics may not have elicited explicit safety disclaimers, or the prompts may have been interpreted as educational rather than as requests for directive clinical advice. Because safety notice coding showed no meaningful interrater agreement, these findings should not be interpreted as evidence that prompt design did or did not influence clinical safety.

Similarly, no refusals occurred in any condition; GPT-5 answered all prompts, including those requesting citations. Although high response compliance may improve usability, it also raises a potential concern: models may provide reference-like material even when those references are not externally verified. This may result in hallucinated or unsupported citations. Given documented concerns about fabricated citations in biomedical contexts, dental educators may wish to request identifiers such as PMIDs or DOIs and independently verify them in PubMed, Crossref, or publisher’s website as part of AI literacy training [26,28,29].

### Implications for Dental Education and Clinical Training

These findings are most directly relevant to how dental educators design, evaluate, and scaffold prompts when using AI-generated content in instructional settings. Because this study examined model outputs rather than learner outcomes, its implications concern the design of AI-supported educational materials rather than the direct effects on student learning or clinical performance.

First, the association between instructional framing and response stance suggests that educators should craft prompts intentionally to reflect the instructional priorities they wish to emphasize, whether patient-centered reasoning, procedural efficiency, reflective analysis, or examination preparation. Because patient-centered prompts did not uniformly produce patient-welfare–oriented responses, educators should not assume that patient priorities will be consistently emphasized unless those priorities are explicitly stated and reinforced.

Second, the effects of evidence requirements must be balanced against their drawbacks. Although evidence-required prompts were associated with higher rater-assessed perceived factuality scores in exploratory analyses and substantially increased citation presence, they also produced longer responses and may introduce pseudoacademic formatting that could confuse learners or obscure essential concepts. An evidence requirement may also alter the apparent certainty of a response, although this study did not establish such an effect. These complexities highlight the need for explicit instructions on how students should interpret, verify, and critically evaluate AI-generated citations.

Third, the low or variable interrater reliability observed for several measures, including perceived factuality and safety notices, underscores the difficulty of evaluating AI outputs consistently, even among trained raters. Consensus scoring does not eliminate this measurement uncertainty. Educators should consider scaffolded training to prepare students and faculty to assess the rigor, clinical appropriateness, and limitations of AI-generated explanations. As AI becomes more integrated into health professions education, the ability to critically appraise model outputs is likely to become an essential professional competency.

### Theoretical Contributions to Prompt Engineering Research

This study contributes to the prompt engineering literature by showing that framing and evidence instructions may be associated with different dimensions of LLM-generated responses. Framing was primarily associated with response perspective, particularly stance orientation, whereas evidence instructions were associated with response structure and citation behavior; an exploratory analysis also suggested an association with perceived factuality. The pattern suggests that different prompt components may influence distinct aspects of AI-generated communication.

The study did not identify statistically significant interactions for most variables. Instead, 2 prompt factors were associated with different response features: framing was associated with rhetorical orientation, whereas evidence requirements were associated with elaboration and citation inclusion. This distinction may guide future work examining how prompt components interact to shape various dimensions of AI-generated pedagogical content.

### Limitations

Several limitations warrant consideration. First, stance orientation exhibited low reliability across raters, suggesting that coding frameworks for complex discourse characteristics require further refinement. Perceived factuality and safety notices also showed low or negative interrater reliability, indicating that these domains were difficult to apply consistently. Although perceived factuality was rated using a structured rubric by trained raters, it did not represent objectively verified clinical accuracy. Therefore, it should be interpreted as rater-assessed perceived accuracy and completeness rather than as an objective measure of factual truth. Similarly, the presence of a safety notice should not be interpreted as a robust indicator of clinical safety. Findings involving these lower-reliability outcomes should be considered exploratory, and consensus scoring does not remove the underlying measurement uncertainty. Intrarater reliability was also not assessed because the study design did not include a repeat-rating phase in which the raters recoded the same responses after a defined interval. Consequently, this study could not determine whether individual raters applied the rubric consistently over time, particularly for interpretive domains such as perceived factuality, safety notices, and stance orientation.

Second, although the factorial design included 40 generated responses, these responses were derived from 10 base prompt topics crossed with 4 experimental conditions. Thus, the study’s conceptual breadth is better understood as 10 prompt topics rather than 40 fully independent prompts. The statistical analyses treated the 40 responses as analytic observations and did not account for the grouping of 4 prompt variants within each base topic. Because responses derived from the same topic shared underlying subject matter, the observations may not have been independent. Failing to account for this clustering may have produced SEs that did not reflect within-topic dependence and may have overstated the precision of the inferential results. In addition, the small number of base topics and sparse categorical events limited statistical power and generalizability. Accordingly, the findings should be interpreted as exploratory, descriptive patterns within this structured prompt set rather than as definitive evidence of broadly generalizable effects.

Third, reliance on the free-tier GPT-5 flagship model limits generalizability to other models, paid versions, or future system updates, which may differ in safety behavior, formatting tendencies, citation behavior, or response style. Because all outputs were generated by a single model through one interface, the findings reflect GPT-5 behavior at the time of data collection rather than prompt effects across all LLM systems. Although each prompt was administered in a new conversation with instructions to disregard prior context, this procedure reduced session-level carryover but did not make the outputs independent of the underlying model system.

Relatedly, the study used the publicly accessible GPT-5 web interface rather than an application programming interface with fixed, reportable generation parameters. Consequently, technical details such as temperature, top-p, hidden system prompts, safety-layer settings, alignment updates, and model snapshot identifiers were unavailable and could not be controlled. Because LLM systems are updated over time, the exact outputs generated in this study may not be fully reproducible in future sessions. To improve transparency, the study reports the data collection procedure in detail and provides sample prompts and responses in Multimedia Appendix 3.

Fourth, citation presence was coded as a structural feature but was not equivalent to citation validity, evidence quality, or the correct interpretation of the cited literature. The study did not verify DOIs or PMIDs, confirm whether each cited reference corresponded to an authentic publication, assess hallucinated references, or evaluate whether the cited literature supported the model’s claims.

Finally, although cavity preparation is a foundational topic in dental training, the study’s narrow clinical-procedural scope limits the generalizability. Because the study examined a single domain, the observed prompt effects may not extend to other areas of dental education, including diagnosis, treatment planning, prevention, patient communication, ethics, or other restorative procedures.

### Future Directions

Future research should investigate how students and faculty use and interpret AI-generated explanations. Studies could examine whether learners mistake unverified or fabricated citations as genuine evidence, how confidence tone influences trust and study behavior, and whether stance orientation affects learning outcomes such as conceptual understanding or clinical reasoning. Future studies should also use larger and more diverse prompt sets across multiple dental education domains, including topics with different levels of evidentiary support. Incorporating validated reference answers, expert-consensus standards, or evidence summaries would allow for more rigorous assessment of factual accuracy and clinical appropriateness.

Additional work should refine and validate coding rubrics for evaluating LLM-generated dental education content. Future studies should assess both interrater and intrarater reliabilities, with intrarater reliability evaluated by having raters recode a subset of responses after a defined interval. This approach would help determine whether individual raters apply the rubric consistently over time, particularly for interpretive domains such as rater-assessed perceived factuality, stance orientation, safety notices, and hedging.

Experiments involving interactive, multiturn dialogue may also reveal more dynamic framing effects as the model adapts to user responses. More sophisticated prompt engineering strategies, such as structured reasoning scaffolds, self-check prompts, reference-verification prompts, and citation-validity checks, could also be tested to determine whether they better align LLM outputs with educational and ethical goals.

### Conclusion

This study found that prompt framing and evidence instructions were associated with systematic differences in the discourse characteristics of GPT-5 responses related to cavity preparation. Evidence requirements were associated with greater citation inclusion, longer responses, and higher rater-assessed perceived factuality scores; instructional framing was associated primarily with response orientation. These findings indicate that prompt wording can influence how LLM-generated educational responses are presented and perceived. As generative AI use expands in dental education, deliberate prompt design may help educators better align AI-generated content with intended instructional objectives. However, objective accuracy, citation validity, clinical safety, and effects on learning require direct evaluation.

## Supplementary material

10.2196/90736 Multimedia Appendix 1 Prompts by framing and evidence requirement.

10.2196/90736 Multimedia Appendix 2 Coding schema.

10.2196/90736 Multimedia Appendix 3 Sample prompts and GPT-5 responses.

## Abbreviations

HIPAA: Health Insurance Portability and Accountability Act
ICC: intraclass correlation coefficient
LLM: large language model
