# Supplementary material for: Prompt Framing and Evidence Requirement for AI-Generated Educational Responses in Dental Education: Experimental Study
Source: JMIR Med Educ. 2026 Aug 6;12:e90736. doi: 10.2196/90736 (PMC13446791; doi:10.2196/90736)
Supplement: Supplementary file 1 [file mededu-v12-e90736-s003.pdf]

Multimedia Appendix 3: Sample prompts and ChatGPT-5 responses.

Base_Topic	Framing	Evidence_Requirement	Prompt_Text	Response_Text

Tooth anatomy essentials for safe cavity preparation	Patient-centered	No evidence	What key aspects of tooth anatomy should dental students focus on when planning and executing cavity preparations to protect the pulp and preserve healthy structure for better patient outcomes?	Great question—this gets at the heart of restorative dentistry. When dental students are planning and executing cavity preparations, they need to balance removing diseased tissue with preserving tooth vitality and function. The key aspects of tooth anatomy to focus on are:<br><br>1. Pulp Anatomy and Protection<br><br>Pulp chamber size and shape: Larger in younger patients, with thinner dentin walls, meaning higher risk of pulp exposure.<br><br>Pulp horns: Extend beneath cusps—especially in anterior teeth and young permanent molars. Over-cutting near cusps increases the risk of pulp exposure.<br><br>Dentin thickness (RDT—Remaining Dentin Thickness): Even 1 mm of dentin provides significant pulpal protection; maintaining maximum RDT reduces postoperative sensitivity.<br><br>2. Enamel Structure<br><br>Enamel rods/prism orientation: Preparations should respect rod direction (e.g., enamel margins supported by dentin) to avoid unsupported enamel fracture.<br><br>Thickness variation: Thicker over cusps, thinner near cervical areas; shallow cervical margins risk exposing dentin and pulp horns.<br><br>Cavo-surface margins: Proper design ensures good bonding/sealing and reduces marginal leakage.<br><br>3. Dentin Properties<br><br>Permeability: Dentin tubules are wider and more numerous closer to pulp—cutting too deep increases risk of bacterial/toxin diffusion.<br><br>Direction of tubules: Tubule density and orientation affect bonding and sensitivity.<br><br>Conservation: Avoid unnecessary removal of sound dentin (especially peri-pulpal dentin) to preserve tooth strength and pulp vitality.<br><br>4. Tooth Morphology

				Occlusal anatomy: Know cusp inclines, grooves, and fossae to design conservative outlines that remove caries but maintain strength.<br><br>Proximal contacts: Preserving contact areas supports periodontal health and prevents food impaction.<br><br>Cuspal support: Overextending into cusps weakens the tooth; consider cusp coverage only if truly necessary.<br><br>5. Biologic Width and Periodontal Considerations<br><br>Be mindful of crown-root anatomy and gingival margins. Preparations extending too far subgingivally risk biologic width invasion, leading to chronic inflammation and bone loss.<br><br>6. Structural Preservation<br><br>Minimize extension: Follow a caries-directed rather than extension-for-prevention approach.<br><br>Rounded internal line angles: Reduce stress concentration and risk of tooth fracture.<br><br>Avoid over-flaring proximal boxes: Prevents unnecessary removal of supporting tooth structure.<br><br>✅ Bottom line:<br>Dental students should focus on maximizing pulpal protection, preserving enamel support, and conserving dentin, all while respecting the natural morphology of the tooth. The goal is a strong, sealed, biologically sound restoration that maintains long-term tooth vitality.<br><br>Would you like me to make you a concise checklist (like a quick-reference for sim clinic) that you can memorize before doing cavity preps?

Tooth anatomy essentials for safe cavity preparation	Patient-centered	Evidence-required	What key aspects of tooth anatomy should dental students focus on when planning and executing cavity preparations to protect the pulp and preserve healthy structure for better patient outcomes? Please cite peer-reviewed dental research.	Here's a tight, evidence-based checklist you can use while planning and cutting preparations. I've grouped the anatomic "must-knows" with what they mean clinically and included peer-reviewed sources for each.<br><br>Enamel & DEJ<br><br>Enamel rod orientation & margin design. Aim to finish margins on supported enamel and (for direct composites on enamel margins) consider a light bevel to expose rod ends and increase bonded enamel area—this consistently reduces microleakage compared with non-beveled margins.<br>PubMed<br>ScienceDirect<br><br>Respect the DEJ as a structural hinge. Over-widened isthmuses and MOD designs raise stress in remaining cusps; smaller preparations preserve stiffness.<br>PMC<br>ScienceDirect<br><br>Dentin: Tubules, Permeability & Remaining Dentin Thickness (RDT)<br><br>Cut orientation & depth with tubules in mind. Closer to the pulp, tubule density/diameter rises sharply—permeability and post-op sensitivity risk go up, and bond behavior changes; keep cuts conservative as you approach the pulp.<br>PubMed<br>PMC<br><br>Protect RDT. Try to maintain ≥0.5–1.0 mm of sound dentin over the pulp whenever possible; thinner RDTs are associated with stronger pulpal reactions, while ≥~0.75–1.0 mm favors reactionary dentin formation instead of exposure.<br>PubMed<br>Wiley Online Library<br><br>Pulp Chamber & Pulp Horns<br><br>Know where the horns are—especially in young teeth. Prominent mesial horns (e.g., maxillary first molars) and generally larger chambers in younger patients increase exposure risk; plan axial/pulpal wall depths accordingly. Aging reduces chamber size and vascularity but doesn't eliminate sensitivity to insult.<br>PMC

				+1<br><br>Marginal Ridges & Cusps<br><br>Save marginal ridges. They're key buttresses; losing both ridges (MOD) or thinning them excessively increases strain and crack risk. Keep isthmuses conservative and avoid over-flaring proximal boxes.<br>PubMed<br>PMC<br><br>Decide early about cuspal coverage. When preparations significantly undermine cusp structure (common in large MODs or endodontically treated/posterior teeth), cuspal coverage improves fracture resistance and clinical longevity versus leaving thin walls.<br>PubMed<br>PMC<br>ScienceDirect<br><br>Caries Removal Strategy Near the Pulp<br><br>Adopt selective caries removal (SCR) for deep lesions. To protect the pulp, stop at soft/leathery dentin over the pulp and create a peripheral seal in sound/firm tissue. RCTs and reviews show SCR maintains vitality and reduces exposure compared with complete excavation or some stepwise protocols, with good 2–5-year outcomes.<br>PMC<br>ScienceDirect<br>BioMed Central<br><br>Internal Form & Stress Management<br><br>Round internal line angles; avoid sharp stress concentrators. This decreases stress in remaining tooth tissue and restorative interfaces, helping limit crack initiation under load. (Shown across FEA/mechanical studies examining cavity geometry and stress.)<br>PMC<br>ScienceDirect<br><br>Functional anatomy matters. Maintain occlusal anatomy and stop-contacts away from thin marginal ridges; improper ridge form concentrates load and promotes fracture and food impaction.<br>PubMed

				Lippincott Journals<br><br>Practical depth targets (rules of thumb, modulated by caries/occlusion)<br><br>Occlusal: keep shallow; rely on adhesive retention rather than "extension for prevention." Aim to stop once you've cleared caries and obtained a peripheral enamel/dentin seal.<br>PMC<br><br>Proximal: break contact minimally and preserve ridge bulk; keep axial wall as shallow as caries allows to preserve RDT over horns. Use SCR if very deep.<br>PubMed<br>PMC<br><br>Why this protects patients<br><br>Preserving enamel support and marginal ridges keeps the tooth stiff and crack-resistant.<br>PMC<br>PubMed<br><br>Maintaining RDT and using SCR lowers pulp inflammation/exposure risk and improves vitality outcomes.<br>PubMed<br>Wiley Online Library<br>PMC<br><br>Respecting tubule/permeability gradients helps you choose adhesives/liners judiciously and anticipate sensitivity.<br>PubMed<br><br>If you'd like, I can turn this into a one-page op-guide you can print for clinic with diagrams of pulp horn locations by tooth and suggested depth "danger zones."

Tooth anatomy essentials for safe cavity preparation	Skill-centered	No evidence	What key aspects of tooth anatomy should dental students master to achieve high accuracy, ideal margins, and consistent grading performance in cavity preparation assessments?	Great question — in cavity preparation, mastering tooth anatomy is what separates technically correct work from truly ideal work that earns consistent grading accuracy. Dental students should focus on the following key aspects:<br><br>1. Pulpal Anatomy and Depth Control<br><br>Pulp chamber and horns: Especially in anterior teeth and young patients, pulp horns extend close to the occlusal surface. Over-reduction risks pulp exposure.<br><br>Dentinal thickness: Maintain at least ~0.5–1 mm of dentin over the pulp for protection.<br><br>Uniform pulpal floor: Flat, smooth, and parallel to the occlusal plane, following external contours without unnecessary depth.<br><br>2. Enamel Rod Orientation<br><br>Margins should terminate on sound enamel supported by dentin. Unsupported enamel fractures easily and leads to poor grading.<br><br>Recognize that enamel rods at the cervical area of permanent teeth incline apically, meaning cervical margins should be slightly internal to avoid leaving unsupported enamel.<br><br>3. Occlusal Morphology<br><br>Primary grooves and pits: Preps should follow natural developmental grooves to conserve tooth structure and blend with anatomy.<br><br>Cuspal inclines: Avoid crossing marginal ridges or weakening cusp tips unless indicated.<br><br>Isthmus width: Keep minimal (usually about 1/4 of intercuspal distance) to preserve cuspal strength and prevent over-extension.<br><br>4. Proximal Surface Relationships<br><br>Contact areas: Breaking contact sufficiently for cleansing but not excessively — ideal for both function and esthetics.<br><br>Gingival margin placement: Smooth, flat, and just below the contact without overextending toward the gingiva.

				Axial wall contour: Should follow the convexity of the external tooth surface, providing uniform dentin support.<br><br>5. Cavosurface Margins<br><br>90° margins in amalgam preps: Prevent marginal breakdown.<br><br>Beveling for composites: Enhances enamel bonding and blending of restorative material.<br><br>Margins should be crisp, smooth, and continuous, with no unsupported enamel, roughness, or irregular extensions.<br><br>6. Tooth-Specific Variations<br><br>Molars vs. premolars: Molar pits and fissures often justify broader occlusal extensions, whereas premolars require more conservative preps.<br><br>Anterior teeth: Pay close attention to lingual anatomy, cingulum thickness, and pulp location when preparing Class III and IV lesions.<br><br>7. Biomechanics and Resistance/Retention Forms<br><br>Internal walls and floors should provide resistance to occlusal forces and prevent displacement of the restorative material.<br><br>Rounded internal line angles reduce stress concentration and enhance prep durability.<br><br>✅ Practical tip for consistent grading:<br>Grading in preclinical courses is often based on precision, symmetry, and respecting natural anatomy. Students who consistently:<br><br>Keep preps centered on grooves/pits,<br><br>Maintain ideal depth (1.5–2 mm for amalgam, 1.5 mm for composite),<br><br>Preserve sound structure while achieving correct extension,<br>tend to score highest and avoid repeat deductions.

				Would you like me to make a step-by-step checklist (like a grading rubric) that you could use during practice to self-assess cavity preparations before submitting them?

Tooth anatomy essentials for safe cavity preparation	Skill-centered	Evidence-required	What key aspects of tooth anatomy should dental students master to achieve high accuracy, ideal margins, and consistent grading performance in cavity preparation assessments? Please cite peer-reviewed dental research.	Here's the tooth-anatomy "short list" that most reliably drives high-accuracy preps, crisp margins, and consistent grades—plus the peer-review behind each point.<br><br>1) Enamel rod orientation → margin strength and finishing<br><br>Rods are not uniformly perpendicular to the surface; their orientation varies by site and depth. Margins cut across rods are weaker and chip more readily. Design cavosurface margins so rod inner ends rest on dentin and outer ends are either supported or fully removed/beveled.<br>thejpd.org<br>PubMed<br>pdf.ipinnovative.com<br><br>Because enamel is anisotropic, cracks propagate more easily transverse to rods—another reason margins that respect rod direction stay cleaner and resist chipping.<br>PMC<br><br>2) The dentino-enamel junction (DEJ) → depth control & unsupported enamel<br><br>The DEJ is a tough, graded interface that arrests/deflects cracks; keeping occlusal depth just into dentin while eliminating undermined enamel preserves this natural "toughening" zone.<br>thejpd.org<br>PMC<br>ScienceDirect<br><br>3) Enamel & dentin thickness by tooth/surface → conservative extension<br><br>Micro-CT studies show enamel thickness varies substantially by tooth and surface (e.g., very thin proximally on mandibular incisors). Translate that into narrower boxes/shallower axial reduction where enamel is thin to avoid over-milling and iatrogenic pulp proximity.<br>PMC<br>MDPI<br>Anatomy Publications<br><br>4) Pulp chamber anatomy & crown dentin thickness → pulp protection<br><br>Contemporary CBCT/µCT work details chamber volumes and roof/floor relations; knowing typical chamber height and horn positions for each tooth type reduces exposures during axial/gingival refinement. (Use radiographs of the specific case to localize horns.)

				MDPI<br>+1<br>PMC<br>dental-research.com<br><br>5) Marginal ridge anatomy → preserve width & height<br><br>Even 1.0–2.0 mm of marginal ridge thickness materially improves fracture resistance; aim to conserve ridge bulk and avoid over-widening isthmuses or proximal boxes.<br>Meridian<br>PubMed<br>Lippincott Journals<br>Dove Medical Press<br><br>6) Proximal contact area (PCA) position/size → contacts that pass checks<br><br>PCA location and apico-incisal extent are tooth-specific and influence papilla fill and contact tightness—key items on grading rubrics. Shape boxes and emerge restorations to re-establish native PCA dimensions and embrasures.<br>PMC<br>go.digitalsmiledesign.com<br><br>Clinical studies show contact "tightness" varies with anatomy/age/tooth position; aim for firm floss "snap" without open or heavy contacts.<br>PMC<br><br>7) Occlusal anatomy (cusp–fossa–groove map) → isthmus placement & wall direction<br><br>Respect primary grooves and cusp inclines so the isthmus stays centered over fissures and axial/box walls parallel the long axis of proximal surfaces—minimizes unsupported enamel and maintains occlusal stops. (Backed by DEJ/rod data above regarding where fractures initiate.)<br>PMC<br>ScienceDirect<br><br>8) Where to place gingival margins → enamel vs. cementum<br><br>When possible, end on enamel for better margin integrity; if a Class II margin must be on cementum, plan for adhesive strategy and contour that restores the cervical emergence

				without over-flaring (reduces marginal leakage/chipping).<br>PMC<br><br>9) Tie anatomy to grading consistency with objective feedback<br><br>Digital comparison systems (e.g., E4D Compare/3D auto-evaluate) improve reliability versus faculty-only grading and help you hit anatomy-driven targets (depth, convergence, margin continuity) the same way every time.<br>PMC<br>PubMed<br>ScienceDirect<br><br>Analytic rubrics also raise inter-rater agreement in clinical operative assessments—use them to translate the anatomy above into checkable criteria (e.g., ridge width preserved, margin on enamel, PCA re-established).<br>BioMed Central<br>+1<br><br>Quick checklist you can apply on every prep<br><br>Rod-respecting margins (no unsupported enamel; clean, continuous cavosurface).<br>thejpd.org<br><br>Depth: into dentin just past DEJ on occlusal; axial/gingival limited by local enamel thickness and pulp map.<br>ScienceDirect<br>PMC<br><br>Preserve marginal ridge bulk (≥1–2 mm thickness when feasible).<br>Meridian<br><br>Isthmus centered over fissure pattern; walls aligned with long axes.<br>PMC<br><br>Re-create native PCA and embrasures; confirm contact tightness clinically.<br>PMC<br><br>Validate with digital comparison + rubric to reduce grading variability.<br>PMC

				BioMed Central<br><br>If you want, I can turn this into a one-page grading rubric keyed to specific tooth types (e.g., maxillary first premolar vs mandibular molar) with target numbers pulled from the cited studies.

Caries detection & outline form planning	Patient-centered	No evidence	How should students use caries detection (visual‑tactile exam, radiographs, adjuncts) to define an outline form that removes disease while preserving tooth structure and minimizing postoperative sensitivity for patients?	Great question — you're essentially asking how dental students should integrate diagnostic tools (visual-tactile exam, radiographs, and adjunctive technologies) into clinical judgment when determining the outline form for caries removal. Here's a structured approach based on operative dentistry and cariology research:<br><br>1. Start with a Caries Risk Assessment<br><br>Before cutting, students should evaluate caries risk, history, and activity.<br><br>A high-risk patient may warrant a slightly more aggressive outline to ensure complete removal, while in a low-risk patient, preservation of enamel and dentin should be prioritized.<br><br>2. Use a Stepwise Caries Detection Approach<br><br>Visual-tactile exam:<br><br>Dry tooth, good lighting, and magnification if available.<br><br>Look for cavitation, color/opacity changes, texture.<br><br>Use an explorer gently to confirm softness (not forcefully, to avoid damaging remineralizable enamel or transmitting bacteria).<br><br>Radiographs:<br><br>Bitewings for proximal lesions, occlusal radiographs for depth estimation.<br><br>Confirm whether lesions are confined to enamel, extend into dentin, or threaten the pulp.<br><br>Adjuncts (e.g., DIAGNOdent, QLF, caries-detecting dyes):<br><br>Useful for borderline cases, but should never override clinical judgment.<br><br>Dyes can over-stain porous dentin, so interpretation must be cautious.<br><br>3. Defining the Outline Form<br><br>Principle: "Remove caries, not tooth." Modern operative dentistry has shifted from GV Black's "extension for prevention" toward minimally invasive dentistry (MID).

				Outline should follow disease location, not arbitrary shapes. For example:<br><br>Occlusal pit/fissure caries: outline only where caries is present, preserve intact fissures when possible.<br><br>Proximal caries: break contact only if needed for caries access, not automatically.<br><br>Maintain strong margins on sound enamel to ensure restoration durability.<br><br>4. Depth and Internal Form Considerations<br><br>Outer infected dentin → remove fully (soft, necrotic, easily scooped).<br><br>Inner affected dentin → may be left if close to pulp, provided it is firm and capable of remineralization.<br><br>Avoid over-deepening the prep to minimize pulpal insult and postoperative sensitivity.<br><br>Round internal line angles to reduce stress concentration and conserve tooth.<br><br>5. Balancing Preservation and Sensitivity<br><br>Overcutting → exposes dentinal tubules, raises risk of sensitivity.<br><br>Undercutting → leaves diseased tissue, risking recurrent caries.<br><br>The ideal balance is achieved by:<br><br>Using caries detection systematically.<br><br>Stopping excavation when dentin is firm, not sticky.<br><br>Preserving pulp vitality via liners or bases only if necessary (deep lesions).<br><br>✅ Key Teaching Takeaway for Students:<br><br>Use diagnostic tools in combination — visual-tactile exam for cavitation, radiographs for depth/location, and adjuncts for borderline lesions. Then, define the outline form strictly

				around carious tissue while preserving as much enamel and dentin as possible, rounding internal angles, and avoiding unnecessary extension. This reduces postoperative sensitivity and maximizes long-term tooth survival.<br><br>Would you like me to also pull in peer-reviewed studies (e.g., on MID, selective caries removal, or the effectiveness of adjuncts like DIAGNOdent) so you can reference them directly in an academic context?

Caries detection & outline form planning	Patient-centered	Evidence-required	How should students use caries detection (visual-tactile exam, radiographs, adjuncts) to define an outline form that removes disease while preserving tooth structure and minimizing postoperative sensitivity for patients? Please cite peer-reviewed dental research.	Here's a concise, evidence-based way to combine visual-tactile findings, bitewings, and adjunctive tools to draw a caries-driven outline form that's conservative and kind to pulps.<br><br>1) Start with a structured visual-tactile exam (ICDAS)<br><br>Use a gentle explorer (or a ball-ended probe) to feel surface texture; avoid forceful probing that can damage demineralized enamel and convert an arrestable lesion into a cavitated one. Document severity with ICDAS so your next steps map to lesion activity.<br>MDPI<br>ScienceDirect<br><br>Implication for outline form:<br><br>ICDAS 1–2 (non-cavitated): no operative outline; plan nonrestorative care (sealants/resin infiltration/fluoride).<br><br>ICDAS 3 with micro-breakdown or clear cavitation (ICDAS 4–6): operative, but keep the prep strictly lesion-driven (no "extension for prevention"). Evidence supports treating early/non-cavitated pits and fissures with sealants to halt progression, avoiding a prep entirely.<br>JADA<br>PMC<br><br>2) Use bitewings to judge cavitation risk and depth<br><br>Radiographs tend to underestimate lesion depth; cavitation likelihood rises with deeper radiolucency, but it's not all-or-none in outer dentin. Inner-dentin radiolucencies have a high probability of cavitation; enamel-only lesions are typically non-cavitated. Consider temporary tooth separation if the decision is uncertain.<br>Semantic Scholar PDFs<br>SciELO<br>MDPI<br><br>Implication for outline form:<br><br>E1–E2 (enamel only): nonrestorative (sealant/infiltration), no outline.<br><br>Outer dentin: combine radiograph + visual (clean, dry, floss) to confirm actual cavitation before prepping; many are still non-cavitated. Keep entry point minimal if you restore.

				Karger<br>MDPI<br><br>3) Adjuncts: use to refine, not replace, your exam<br><br>Adjuncts (e.g., laser fluorescence/impedance, QLF/FOTI) can raise sensitivity in some contexts, but they don’t consistently outperform visual+radiographic methods and should not lower your operative threshold by themselves. Use them to monitor or to investigate uncertain sites.<br>PMC<br>+1<br>SAGE Journals<br>Nature<br><br>4) Define a conservative, caries-driven outline form<br><br>Follow minimal-intervention principles: access only to remove active, infected dentin and to create conditions for durable adhesion; do not prophylactically extend to “self-cleansing” areas.<br>PubMed<br><br>Peripheral enamel/dentin: remove to sound, hard margins; preserve enamel where supported and bondable.<br><br>Pulpal/axial floors in deep lesions: use selective caries removal (leave firm/affected dentin near the pulp) rather than complete excavation—this reduces pulp exposures without harming outcomes. If very deep, consider stepwise excavation.<br>PMC<br>+1<br><br>For non-cavitated occlusal/proximal lesions: prefer sealants or resin infiltration over operative care to preserve tooth structure.<br>JADA<br>PubMed<br><br>5) Minimize postoperative sensitivity (biology + bonding)<br><br>Preserve remaining dentin thickness (RDT): thinner dentin and opened tubules ↑ dentin permeability and pulpal response. Avoid over-drying and maintain/replace the smear when

				appropriate.<br>PMC<br>staging.aapd.org<br><br>Adhesive strategy: when used correctly, self-etch vs total-etch shows no consistent difference in sensitivity; meticulous technique (isolation, proper etch time if used, solvent/air management, full curing) matters more than the category.<br>PMC<br>PubMed<br><br>Liners/Bases: routine liners under composites do not reduce postoperative sensitivity and aren't needed in shallow/moderate cavities. Reserve RMGI as a thin liner for very deep areas to block out and for adhesion on sclerotic dentin as needed; for near exposures or actual pinpoint exposures, use calcium-silicate (MTA/Biodentine) for pulp protection per vital pulp therapy evidence.<br>PMC<br>+1<br>Lippincott Journals<br><br>6) A simple chairside algorithm<br><br>Visual-tactile (ICDAS) + bitewings → decide cavitated vs non-cavitated. Use adjuncts only if uncertainty persists.<br>ScienceDirect<br>MDPI<br><br>Non-cavitated (E1–E2 / early D): nonrestorative—sealant/infiltration/fluoride and risk-based prevention; no prep.<br>JADA<br>PubMed<br><br>Cavitated or cleansable defect not maintainable: prep only enough for access, debridement, and reliable bonding; keep margins on sound tissue; avoid unnecessary cusp/marginal ridge removal. Minimal-intervention principles apply.<br>PubMed<br><br>Deep dentin: perform selective caries removal (leave affected dentin pulpal/axial); if pulp is nearly exposed/exposed, place calcium-silicate liner/cap and restore.<br>PMC

				Lippincott Journals<br><br>Bond thoughtfully: isolate, avoid dentin overdrying, follow adhesive IFU; don’t rely on routine liners to prevent sensitivity.<br>PMC<br>+1<br><br>Key sources (peer-reviewed)<br><br>Selective/incomplete caries removal: Cochrane & systematic reviews show fewer exposures and comparable outcomes vs complete excavation.<br>PMC<br>+1<br><br>ICDAS & visual-tactile reliability: systematic reviews/meta-analyses support structured visual criteria to stage disease.<br>ScienceDirect<br><br>Radiographic depth vs cavitation: enamel radiolucencies are commonly non-cavitated; inner-dentin lesions are often cavitated; radiographs underestimate depth—use with clinical confirmation.<br>Semantic Scholar PDFs<br>SciELO<br>MDPI<br><br>Nonrestorative options: sealants prevent and arrest early lesions; resin infiltration arrests many non-cavitated proximal lesions.<br>JADA<br>PubMed<br><br>Adjuncts: adjunct technologies add information but don’t outperform the combined visual+radiographic approach for treatment decisions.<br>PMC<br>+1<br><br>Postop sensitivity & pulp protection: permeability and RDT govern risk; adhesive category alone doesn’t determine sensitivity; routine liners don’t help; calcium-silicates are effective for VPT.<br>PMC

				+2<br>PMC<br>+2<br>Lippincott Journals<br><br>If you’d like, I can turn this into a one-page clinic checklist for students.

Caries detection & outline form planning	Skill-centered	No evidence	How should students use caries detection findings to plan an outline form that meets common grading rubrics for extension, retention, and conservation in simulation exams?	Great question — you're asking about how students can translate caries detection findings into an outline form that not only follows clinical principles but also matches the way simulation exams are graded. In sim clinic, the challenge is balancing real-world conservation of tooth structure with the standardized expectations of faculty grading rubrics.<br><br>Here's a framework students can follow:<br><br>1. Extension: Defining the Outline Form<br><br>Base it on caries detection findings<br><br>Use visual-tactile exam and radiographs (if provided in sim) to identify actual carious dentin/unsupported enamel in the preparation area.<br><br>Extend only enough to eliminate caries and unsupported enamel, but in exams, faculty expect you to "break contacts" and include the classic extension criteria (e.g., proximal box to just clear contact, occlusal walls extending to the central fissures).<br><br>Rubric focus: Examiners look for smooth, continuous outline form with no unsupported enamel, margins placed on cleansable, caries-free tooth structure.<br><br>2. Retention: Internal Form and Wall Convergence<br><br>Caries detection findings tell you where the lesion penetrates, which helps decide the depth.<br><br>Remove infected dentin while preserving affected dentin near the pulp when possible.<br><br>Keep the pulpal and gingival floors flat and on sound dentin.<br><br>Faculty grading rubrics often emphasize:<br><br>Proper wall convergence (typically 6–10° for amalgam preps).<br><br>Adequate depth for material retention (≈1.5–2.0 mm in occlusal).<br><br>Resistance/retention form that would realistically support the restoration.<br><br>3. Conservation: Preserving Healthy Tooth Structure

				Caries detection helps distinguish diseased vs. sound enamel/dentin.<br><br>In exams, over-extension is often penalized just as much as under-extension.<br><br>Strategies:<br><br>Keep the preparation within natural grooves and pits when possible.<br><br>Use the "ideal" prep dimensions taught in lecture as guidelines, but adapt slightly if simulated caries dictates it.<br><br>Avoid unnecessary removal of cusps or marginal ridges unless clearly undermined.<br><br>4. Practical Exam Strategy<br><br>Start with a mental blueprint: outline based on both lesion location and ideal prep dimensions from the rubric.<br><br>Follow the sequence: caries detection → ideal outline → smooth refinement.<br><br>Check against rubric language:<br><br>Extension: margins clear of caries/contacts, smooth curves.<br><br>Retention: proper convergence and depth.<br><br>Conservation: minimal but sufficient extension, no "over-carving."<br><br>✅ Summary for students:<br>Use caries detection to decide where extension is biologically necessary (disease removal), but shape and refine your outline to match the grading rubric's expectations for extension (cleanable margins), retention (wall form and depth), and conservation (avoid over-extension). In exams, the "sweet spot" is showing you can remove disease logically while still demonstrating mastery of the ideal prep form faculty want to see.<br><br>Would you like me to make you a checklist version of this (almost like a step-by-step scoring guide you could use before turning in a prep in sim)?
