# Supplementary material for: Prompt Framing and Evidence Requirement for AI-Generated Educational Responses in Dental Education: Experimental Study
Source: JMIR Med Educ. 2026 Aug 6;12:e90736. doi: 10.2196/90736 (PMC13446791; doi:10.2196/90736)
Supplement: Supplementary file 2 [file mededu-v12-e90736-s001.docx]

**Multimedia Appendix 1: Prompts by framing and evidence requirement.**

| **Topic Category** | **Framing** | **Evidence Requirement** | **Prompt Text (Verbatim)** |
| --- | --- | --- | --- |
| Tooth Anatomy & Patient Experience | Patient-centered | No evidence | What key aspects of tooth anatomy should dental students focus on when planning and executing cavity preparations to protect the pulp and preserve healthy structure for better patient outcomes? |
| Tooth Anatomy & Patient Experience | Patient-centered | Evidence-required | What key aspects of tooth anatomy should dental students focus on when planning and executing cavity preparations to protect the pulp and preserve healthy structure for better patient outcomes? Please cite peer-reviewed dental research. |
| Tooth Anatomy & Academic Performance | Skill-centered | No evidence | What key aspects of tooth anatomy should dental students master to achieve high accuracy, ideal margins, and consistent grading performance in cavity preparation assessments? |
| Tooth Anatomy & Academic Performance | Skill-centered | Evidence-required | What key aspects of tooth anatomy should dental students master to achieve high accuracy, ideal margins, and consistent grading performance in cavity preparation assessments? Please cite peer-reviewed dental research. |
| Caries Detection & Patient Experience | Patient-centered | No evidence | How should students use caries detection (visual-tactile exam, radiographs, adjuncts) to define an outline form that removes disease while preserving tooth structure and minimizing postoperative sensitivity for patients? |
| Caries Detection & Patient Experience | Patient-centered | Evidence-required | How should students use caries detection (visual-tactile exam, radiographs, adjuncts) to define an outline form that removes disease while preserving tooth structure and minimizing postoperative sensitivity for patients? Please cite peer-reviewed dental research. |
| Caries Detection & Academic Performance | Skill-centered | No evidence | How should students use caries detection findings to plan an outline form that meets common grading rubrics for extension, retention, and conservation in simulation exams? |
| Caries Detection & Academic Performance | Skill-centered | Evidence-required | How should students use caries detection findings to plan an outline form that meets common grading rubrics for extension, retention, and conservation in simulation exams? Please cite peer-reviewed dental research. |
| Equipment Selection & Patient Comfort | Patient-centered | No evidence | What strategies should students use to select burs and hand instruments for cavity preparation to reduce vibration, heat, and patient discomfort while maintaining safe, controlled cutting? |
| Equipment Selection & Patient Comfort | Patient-centered | Evidence-required | What strategies should students use to select burs and hand instruments for cavity preparation to reduce vibration, heat, and patient discomfort while maintaining safe, controlled cutting? Please cite peer-reviewed dental research. |
| Equipment Selection & Academic Performance | Skill-centered | No evidence | What strategies should students use to select burs and hand instruments for cavity preparation to optimize cutting efficiency and achieve ideal internal form and finish in practical exams? |
| Equipment Selection & Academic Performance | Skill-centered | Evidence-required | What strategies should students use to select burs and hand instruments for cavity preparation to optimize cutting efficiency and achieve ideal internal form and finish in practical exams? Please cite peer-reviewed dental research. |
| Ergonomics & Patient Experience | Patient-centered | No evidence | Which ergonomic positions and hand-stabilization techniques during cavity preparation best minimize slips and trauma, promoting gentle and safe treatment for patients? |
| Ergonomics & Patient Experience | Patient-centered | Evidence-required | Which ergonomic positions and hand-stabilization techniques during cavity preparation best minimize slips and trauma, promoting gentle and safe treatment for patients? Please cite peer-reviewed dental research. |
| Ergonomics & Academic Performance | Skill-centered | No evidence | Which ergonomic positions and hand-stabilization techniques help students maintain precision, speed, and consistency during cavity preparation to maximize exam performance? |
| Ergonomics & Academic Performance | Skill-centered | Evidence-required | Which ergonomic positions and hand-stabilization techniques help students maintain precision, speed, and consistency during cavity preparation to maximize exam performance? Please cite peer-reviewed dental research. |
| Isolation & Patient Experience | Patient-centered | No evidence | How should students choose and apply isolation and moisture control (e.g., rubber dam, cotton rolls, suction) during cavity preparation to improve patient safety, comfort, and long-term restorative outcomes? |
| Isolation & Patient Experience | Patient-centered | Evidence-required | How should students choose and apply isolation and moisture control (e.g., rubber dam, cotton rolls, suction) during cavity preparation to improve patient safety, comfort, and long-term restorative outcomes? Please cite peer-reviewed dental research. |
| Isolation & Academic Performance | Skill-centered | No evidence | How should students choose and apply isolation and moisture control during cavity preparation to achieve predictable field control and ideal margins that meet grading standards? |
| Isolation & Academic Performance | Skill-centered | Evidence-required | How should students choose and apply isolation and moisture control during cavity preparation to achieve predictable field control and ideal margins that meet grading standards? Please cite peer-reviewed dental research. |
| Infection Control & Patient Safety | Patient-centered | No evidence | What infection control steps should students follow before, during, and after cavity preparation to protect patients from cross-contamination and aerosol risks? |
| Infection Control & Patient Safety | Patient-centered | Evidence-required | What infection control steps should students follow before, during, and after cavity preparation to protect patients from cross-contamination and aerosol risks? Please cite peer-reviewed dental research. |
| Infection Control & Academic Performance | Skill-centered | No evidence | What infection control steps should students follow around cavity preparation to meet clinical requirements efficiently and avoid deductions in practical assessments? |
| Infection Control & Academic Performance | Skill-centered | Evidence-required | What infection control steps should students follow around cavity preparation to meet clinical requirements efficiently and avoid deductions in practical assessments? Please cite peer-reviewed dental research. |
| Cavity Preparation & Long-term Outcomes | Patient-centered | No evidence | How should students apply minimally invasive design principles during cavity preparation to preserve sound enamel/dentin and support long-term tooth health for patients? |
| Cavity Preparation & Long-term Outcomes | Patient-centered | Evidence-required | How should students apply minimally invasive design principles during cavity preparation to preserve sound enamel/dentin and support long-term tooth health for patients? Please cite peer-reviewed dental research. |
| Cavity Preparation & Academic Performance | Skill-centered | No evidence | How should students apply minimally invasive design principles during cavity preparation to achieve ideal dimensions with efficient cutting and strong assessment scores? |
| Cavity Preparation & Academic Performance | Skill-centered | Evidence-required | How should students apply minimally invasive design principles during cavity preparation to achieve ideal dimensions with efficient cutting and strong assessment scores? Please cite peer-reviewed dental research. |
| Use of Liners & Patient Experience | Patient-centered | No evidence | How should students control preparation depth and decide when to place liners or bases to minimize risk of pulpal injury and postoperative sensitivity for patients? |
| Use of Liners & Patient Experience | Patient-centered | Evidence-required | How should students control preparation depth and decide when to place liners or bases to minimize risk of pulpal injury and postoperative sensitivity for patients? Please cite peer-reviewed dental research. |
| Use of Liners & Academic Performance | Skill-centered | No evidence | How should students control preparation depth and select liners/bases when indicated to meet dimensional tolerances and criteria in grading rubrics? |
| Use of Liners & Academic Performance | Skill-centered | Evidence-required | How should students control preparation depth and select liners/bases when indicated to meet dimensional tolerances and criteria in grading rubrics? Please cite peer-reviewed dental research. |
| Iatrogenic Damage & Patient Safety | Patient-centered | No evidence | What techniques help students prevent iatrogenic damage to adjacent teeth and soft tissue during proximal access and finishing, reducing harm and improving patient safety? |
| Iatrogenic Damage & Patient Safety | Patient-centered | Evidence-required | What techniques help students prevent iatrogenic damage to adjacent teeth and soft tissue during proximal access and finishing, reducing harm and improving patient safety? Please cite peer-reviewed dental research. |
| Iatrogenic Damage & Academic Performance | Skill-centered | No evidence | What techniques help students avoid nicking adjacent surfaces and over-extension during proximal access so they retain contacts and avoid point deductions in practical exams? |
| Iatrogenic Damage & Academic Performance | Skill-centered | Evidence-required | What techniques help students avoid nicking adjacent surfaces and over-extension during proximal access so they retain contacts and avoid point deductions in practical exams? Please cite peer-reviewed dental research. |
| Student Explanation & Patient Experience | Patient-centered | No evidence | How should students explain a cavity preparation, including steps, risks, benefits, and aftercare, to reduce patient anxiety and support informed consent? |
| Student Explanation & Patient Experience | Patient-centered | Evidence-required | How should students explain a cavity preparation, including steps, risks, benefits, and aftercare, to reduce patient anxiety and support informed consent? Please cite peer-reviewed dental research. |
| Student Explanation & Academic Performance | Skill-centered | No evidence | How should students structure a cavity-preparation explanation to efficiently cover required points and perform well on communication OSCEs and clinical assessments? |
| Student Explanation & Academic Performance | Skill-centered | Evidence-required | How should students structure a cavity-preparation explanation to efficiently cover required points and perform well on communication OSCEs and clinical assessments? Please cite peer-reviewed dental research. |
