# Supplementary material for: Prompt Framing and Evidence Requirement for AI-Generated Educational Responses in Dental Education: Experimental Study
Source: JMIR Med Educ. 2026 Aug 6;12:e90736. doi: 10.2196/90736 (PMC13446791; doi:10.2196/90736)
Supplement: Supplementary file 3 [file mededu-v12-e90736-s002.docx]

**Multimedia Appendix 2: Coding Schema.**

| Variable | Type | Scale / Values | Description |
| --- | --- | --- | --- |
| Factuality | Ordinal (0–3 scale) | 0 = Factually incorrect or missing critical content  1 = Partially accurate with notable gaps  2 = Mostly accurate with minor omissions  3 = Accurate and clinically complete | Captures accuracy and completeness of clinical content based on rater education and clinical experience. |
| Confidence Tone | Ordinal (0–3 scale) | 0 = Highly hedged or uncertain throughout  1 = Predominantly hedged with some assertiveness  2 = Mostly assertive with some hedging  3 = Fully assertive and confident throughout | Assesses assertiveness, certainty, and hedging in the response's tone. |
| Stance Orientation | Categorical | **Patient-welfare oriented**  Focused on the patient's well-being: comfort, safety, long-term health, and overall experience.  **Performance-oriented**  Focused on the learner's performance: technical skills, efficiency, and success on evaluations.  **Neutral/mixed**  Feedback emphasizing both patient-welfare and practitioner-performance. | Classifies responses as patient-welfare oriented, performance-oriented, or neutral/mixed. |
| Citations Present | Binary | 0 = Absent 1 = Present | Codes whether the model included reference-like citations (not necessarily verified). |
| Safety Notice Flag | Binary | 0 = Absent 1 = Present | Identifies the presence of warnings, disclaimers, or safety caveats. |
| Refusal Flag | Binary | 0 = Absent 1 = Present | Identifies whether the model declined to answer the prompt. |
| Hedging Count | Frequency | Integer count (≥ 0) | Tallies hedging terms such as "might," "could," and "possibly." |
| Response Length | Computed Outcome | Character count (integer) | Character count obtained programmatically from the full response text. |
